# Chronic Endocarditis without Acute Onset

**Published:** 1919-01

**Authors:** 


**Chronic Endocarditis without Acute Onset.** By L. M. WARFIELD, Captain M. R. C. *Journal of the American Medical Association*, Sept. 2, 1918.

The author states that, owing to the medical examination of thousands of men for the army, and to the fact that diseases can, at present, in many cases be observed in hospital from their very beginning, some of our ideas on the influence of certain infectious diseases in the development of chronic valvular heart disease have been confirmed, while others have had to be modified.

There follows a statistical study of heart lesions, mitral stenosis, aortic and mitral insufficiency based upon observation of men at the Recruit Depot, Jefferson Barracks, Mo. The author first noted that men were frequently discharged from the army on account of mitral stenosis, who showed absolutely normal hearts. All cases reported as mitral stenosis were then re-examined and classified as such only when showing definite symptoms: presystolic thrill, presystolic rumble ending in a snappy first sound. The left ventricle was in no case enlarged.

In some cases, no infection to which the disease of the heart valve might be imputed could be recorded; only in the case of a few older men could the disease be attributed to syphilis.

The table below is based upon the examination of 43,427 men between the ages of 18 and 39, among whom 24,002 were drafted men and 19,425 volunteers.

| | Total number of cases. |
|---|---|
| Heart lesions | 157 |
| Infectious diseases | 95 |
| Mitral stenosis | 29 (1) |
| Aortic insufficiency | 22 (2) |
| Mitral insufficiency | 106 (3) |

(1) No history of infection in 8 cases.
(2) No history of infection in 12 cases.
(3) No history of infection in 42 cases.

It must be noted, however, that in some cases men can give no history of infection, although infection has existed in early childhood. On the other hand, not only are these cases rare, but it is doubtful whether infection in infancy can influence the development of chronic valvular disease to the same degree as infections occurring in later life.

Cases of mitral stenosis and aortic insufficiency were caused by changes in the valves leading to shortening, thickening, and adhesions. Owing to the varieties of systolic murmurs heard at the apex, many of which are but functional, it was often impossible to determine the organic or functional cause in cases of mitral insufficiency. Syphilis played but a small part in cases of aortic insufficiency. It must be remembered, however, that most of the men upon whom this study was based were under 25 years of age, and that aortitis of syphilis is usually found in men after 30, in most cases after 40.

The author believes that, in cases where there was no history of infection, infection had existed in a state sufficiently mild to have escaped the notice of the patient, but strong enough to have caused damage to the valves. He assumes that there are cases of mild, symptomless endocarditis as there are cases of "walking typhoid", mild pneumonia, etc. Such cases are found accidentally, when the supposedly healthy man is examined.

